# Big data insight on global mobility during the Covid-19 pandemic lockdown

**DOI:** 10.1186/s40537-021-00474-2

**Published:** 2021-06-02

**Authors:** Adam Sadowski, Zbigniew Galar, Robert Walasek, Grzegorz Zimon, Per Engelseth

**Affiliations:** 1 Faculty of Economics and Sociology, Institute of Logistics and Informatics, University of Lodz, Rewolucji 1905 r. 37, 90-214 Lodz, Poland; 2 Barry Callebaut SSC Europe Sp. Z O.O., Wolczanska 180, 90-530 Lodz, Poland; 3 Department of Management, Jan Kochanowski University in Kielce, Zeromskiego Street 5, 25-369 Kielce, Poland; 4 Department of Finance, Banking and Accounting, Rzeszow University of Technology, Al. Powstancow Warszawy 12, 35-959, Rzeszow, Poland; 5 Tromsø School of Business and Economics, UiT The Arctic University of Norway, Narvik Campus, Lodve Langes gate 2, 8514 Narvik, Norway

**Keywords:** Human mobility, Covid-19, Lockdown, Big data, Human dynamics, Correlation

## Abstract

The Covid-19 pandemic that began in the city of Wuhan in China has caused a huge number of deaths worldwide. Countries have introduced spatial restrictions on movement and social distancing in response to the rapid rate of SARS-Cov-2 transmission among its populations. Research originality lies in the taken global perspective revealing indication of significant relationships between changes in mobility and the number of Covid-19 cases. The study uncovers a time offset between the two applied databases, Google Mobility and John Hopkins University, influencing correlations between mobility and pandemic development. Analyses reveals a link between the introduction of lockdown and the number of new Covid-19 cases. Types of mobility with the most significant impact on the development of the pandemic are “retail and recreation areas", "transit stations", "workplaces" "groceries and pharmacies”. The difference in the correlation between the lockdown introduced and the number of SARS-COV-2 cases is 81%, when using a 14-day weighted average compared to the 7-day average. Moreover, the study reveals a strong geographical diversity in human mobility and its impact on the number of new Covid-19 cases.

## Introduction

Since the first case of SARS-Cov-2 infection was detected and confirmed in China, there has been a huge increase in the number of confirmed cases worldwide. As of March 20, 2021, there were 121,759,000 confirmed cases worldwide, including 2,690,731 deaths, according to data of World Health Organization (WHO). As of 18 March 2021, a total of 364,184,603 vaccine doses have been administered. The significant scale of infections and the rapid spread of the virus in the environment have led the WHO to consider this epidemic to be a pandemic that has affected the entire international community. Despite the efforts of various international and national organizations, including pandemic governments, medical services, medical laboratories, universities and research centers, so far it has proved impossible to significantly limit the increase of Covid-19 cases. For this reason, understanding the existing relationship between the spread of the virus and the mobility of people is crucial to mitigate the global negative effects of the pandemic from the perspective of society and the world economy.

The first studies were conducted in countries affected by the Covid-19 pandemic, such as China, South Korea and Italy. The governments’ restrictions introduced in these countries in the form of lockdown, contact tracing and quarantine have reduced the number of people infected. However, the global nature of the pandemic requires a broad view of the problem of virus transmission using big data for all world regions and countries. Our research is the first to explain the relationship between changes in mobility and the spread of the Covid-19 pandemic from a global perspective.

Since the onset of the Covid-19 pandemic in January 2020, few research results have been available to explain the relationship between human mobility and Covid-19 transmission using a variety of sources and datasets. Initially, as we mentioned, these studies were conducted in countries that were the first to face the specter of the uncontrolled Covid-19 spread. These studies mainly used national data sources on mobility changes in different space–time systems, relating to regions and cities in a given country. For example, a study conducted in China by Haimeng Liu, Chuanglin Fang and Qian Gao was based on the Health Index of Cities (HIC) using two types of human mobility data from Baidu to construct the HIC. Baidu is one of the largest map service providers in China. In contrast, confirmed COVID-19 cases come from the National Health Commission of China (http://www.nhc.gov.cn/xcs/yqtb/list_gzbd.shtml ) [1]. A similar approach based on national data sources was used by Marino Gatto et al. [2] in a study conducted in Italy which estimated the parameters of metacommunity Susceptible-Exposed-Infected-Recovered (SEIR)-like transmission model. The study covered 107 provinces connected by mobility. The model includes sequential restrictions introduced by the Italian Government broken down by provinces, which resulted in partial or complete lockdown and mobility restrictions. Similar, space-oriented national or regional study results using human mobility data are also available for places such as the USA, Japan, China or Hong-Kong [3–8].

Previous studies have limitations that do not permit a comprehensive look at the impact of changes in different types of mobility on the development of the Covid-19 pandemic worldwide. First of all, the conclusions are drawn on the basis of a short period covering the first months since the beginning of the pandemic, e.g. [9], which refers to the lockdown of spring 2020. Some are focused on exploring the interdependencies of government measures under the Covid-19 anti-transmission policy [10]. Others used the Oxford COVID-19 Government Response Tracker (OxCGRT) database COVID-19 and The Global Health Security Index (GHSI) as the basis for the formulation of policy responses [11, 12]. However, these research approaches do not provide a full opportunity to demonstrate the relationship between changes in mobility and the development of the current pandemic.

For the first time in the history of mobility research, thanks to the availability of data, it is possible to show the impact of global phenomena, such as a pandemic, on global mobility. In our research, we have adopted a big data approach to illustrate the relationship between lockdown and mobility, which lifts any limitations in the spatial scope of the analysis.

The use of a data integration approach in epidemiological research has a long history. A common approach is applying the Bradford Hill Criteria (strength of association, consistency, specificity, temporality, biological gradient, plausibility, coherence, experiment, and analogy) to determine causality in exposure–response associations in human disease [13]. However, our research is primarily aimed at determining whether there is a relationship between the development of the Covid-19 pandemic and mobility without clearly indicating the cause-and-effect nature of the relationship.

The contribution of our research to the current scientific discourse is threefold. Firstly, the time range adopted for the study covers the full period from the beginning of the pandemic to the start of the first vaccination campaign in the UK. Secondly, the analyses conducted and conclusions regarding the impact of mobility changes in different areas of activity concern 137 countries and 50 U.S. states included in the Google Mobility survey, which gives a global perspective on the possible effects of lockdown on regional, national and smaller spatial unit level (Appendix A, Table 5). Thirdly, our contribution lies in the in-depth presentation of methodologies for the processing and integration of databases, which can also be used in further research on Covid-19. The conceptual model used in the studies assumes a correlation between different types of mobility and SARS-Cov-2 transmission.

This has also given a dynamic view of the mobility changes caused by the lockdown. The contribution of this research lies in its global perspective on the relationship between mobility and the spread of Covid-19. This requires the use of data that describe all countries, which allows comparisons and gives rise to the initial generalization of the results obtained. The results of our research are universal and can be used at the level of various international, national and regional institutions to assess and revise the existing lockdown policies.

The Covid-19 pandemic, which caused the global lockdown, has greatly affected changes in mobility around the world, regardless of the country and region's position in the global labor division. In our research, we have shown the extent to which the Covid-19 pandemic has influenced changes in global mobility. In different countries, governments have adopted different approaches to lockdown, from liberal ones, like in Italy and Sweden, to very restrictive ones, like in China. It is worth noting that the mobility restrictions are introduced in each country in a different way—most often, gradually, depending on the changes in the number of Covid-19 active cases. However, regardless of the nature of the lockdown, there have been significant changes in public activity and total mobility in all countries.

In this study, we have used cellular data from Google on six types of mobility resulting from the need to maintain social distance. These data were made public during the Covid-19 pandemic period. Google data include: retail & recreation areas, groceries & pharmacies, parks, transit stations, workplaces and residential areas. They allow for an in-depth dynamic analysis of mobility changes across these categories in the context of the Covid-19 global pandemic. We use these data to consider the effect this lockdown and mobility limitations have had on the number of Covid-19 cases.

This research analyzes the effect of the lockdowns in the studied countries in regards of type of lockdown. It directs attention to the organizational setting of lockdown as *societally organized locations*: retail & recreation areas, groceries & pharmacies, parks, transit stations, workplaces and residential areas. Furthermore, lockdown is administered over time, and the feature of timing the lockdown in the number of days is also considered.

Fundamentally, an inductive approach is applied based on the assumptions of the grounded theory where a theory is systematically generated from data [14]. Due to the lack of studies on links between mobility and the Covid-19 pandemic, we have described our approach as loose and emerging, which fosters active testing of all possible interdependencies. However, the studies carried out are quantitative and based on many algorithms that have been used to process the data. Due to the lack of possibility to pre-determine the quality of the results of the integration of the Google Mobility and John Hopkins University databases, the grounded theory provides an opportunity to modify the theoretical framework of the research [15]. The following literature review provides an overview of the Covid-19 impact on global mobility followed by a section covering the applied approach on Big Data in mobility studies.

The structure of this paper consists of five parts apart form the introduction. In Sect. "Literature review/theoretical background" the theoretical background of the study is introduced. This section offers an in-depth discussion on the impact of Covid-19 on global mobility and Big data's approach to mobility studies. The next Sect. "Research methodology" presents the applied methodology. This involves using the Covid-19 John Hopkins University database and Google Mobility database as grounds for analyzing. Section "Results" describes combining data into the Mobility-Covid-19 database and human mobility impact on Covid-19 transmission. In Sect. 5 findings in the form of results are presented. In the final section general conclusions and further research directions are briefly discussed. The appendix provides the more detailed empirical findings.

## Literature review/theoretical background

### Covid-19 impact on global mobility

Global mobility is characterized by relatively constant patterns of human behavior across countries, regions and cities. This situation relates to periods of social and economic stability not disturbed by economic crises, social conflicts, wars, natural and technological disasters and other phenomena which give rise to changes in mobility [16]. The impact of factors affecting mobility varies greatly and is most often limited to countries and regions, as in the case of natural disasters such as the tsunami in Japan, the earthquakes in Turkey, or the MERS in the Middle East and SARS pandemics in Wuhan [17]. It is extremely rare to see factors affecting mobility have a global reach and strike all countries and regions at the same time, causing a simultaneous global lockdown of societies and economies.

The theoretical framework of our research refers to the paradigm of mobilities proposed by Urry to study social phenomena related to human activity in the field of movement [18–20]. The first theoretical trend to which we refer in our article concerns the specificities and patterns in the theory of mobility across countries and regions, which directly affected the rate and directions of the spread of Covid-19. The originality of our research consists in dynamic tracking of the changes in global mobility across internal territorial units such as counties, states, countries, regions, and, eventually, the entire world. The second theoretical trend is related to the use of big data in the area of mobility to track changes in people’s movement patterns. This trend is associated with human dynamics. Pandemics are phenomena that have affected current global mobility, causing significant changes in the behavior of the world community [21]. While studying them, it is possible to use a location-based approach to monitor urban movements [22–24]. This approach can be used on a broader scale to include changes in mobility at national and regional levels.

In our research, we agree with van Cranenburgh [25] that *a substantial change is an unconventional change that directly or indirectly causes an "enduring" change in at least one principal indicator of mobility at least 5% on a supranational scale*. According to substantial typology of changes presented in the study of van Cranenburgh (2012), Covid-19 is classified into the category of disasters (abrupt substantial changes in the biosphere), just as SARS, MERS, H1N1, avian flu or swine flu were in the past. However, given the level and geographical scope of impact and the effect on society and the economy, we should consider whether the 5% level of change can determine the relevance of future mobility changes.

Spatial mobility is contemplated on the basis of various research trends and related theoretical concepts [26, 27]. In the area of sociology, Urry (2007) gives definitions of types of mobility calling them simply *mobilities*, and links them to social life in late modernity. He distinguishes various types among them, such as medical travel, visiting friends and relatives, work-related travel including commuting, 'trailing travel' of children, partners, other relatives and domestic servants. These types are the quickest to respond to disruptions in the form of disasters such as Covid-19. Others, such as tourism travel, react to social distancing and lockdown caused by the pandemic at a slower pace [28, 29].

Sheller (2016) points to the interdependence between disease mobilities and transportation, indicating deeper consequences for countries and regions, triggered by the spread of the epidemic from a historical perspective [30]. Disease mobilities are understood as more-than-human mobilities, which requires the adoption of a research approach that will capture the mechanism of transmission of infectious diseases [31]. The spread of Covid-19 differs significantly from previous epidemics in both transmission rate and geographical scope. The problem also lies in the asymptomatic nature of many cases, which affects the fluctuations in the number of confirmed cases and the validity of the decision on lockdown.

However, after a certain period into the pandemic, all mobility, regardless of its type, changes, leading to a shift in its valorization by the society. Work-related travel is replaced by remote work, which leads to a restriction of the movement of employees.

Few partial results from studies on changes in spatial mobility caused by Covid-19 indicate that Italy saw a decrease in mobility by around 50% between March 10 and April 1 [32]. The studies cited were used as a research tool for an application originally designed to provide real-time seismic monitoring on a sample of 20,000 Italian app users. These and similar research results at a national level do not permit a global view of the actual impact of Covid-19 on mobility. Moreover, due to time constraints, human dynamics associated with the reaction to lockdown cannot be illustrated.

Changes in human mobility caused by the Covid-19 pandemic also stem from recent research on policy stringency and trust for governments of countries that introduced a total or partial lockdown [33].

### Big data in mobility studies

Thanks to the use of big data, studies on mobility are changing in nature and vary from those carried out in the past. This applies to both increasing the scope of spatial analyses and the volume of data that can be analyzed in real time. This leads to an increased number of quantitative studies that use algorithms to enable in-depth analysis of GPS data from mobile phone users [34]. We believe, similarly to Brockmann [35], that the understanding of *human mobility and the development of qualitative models as well as quantitative theories for it is of key importance in the research of human infectious disease dynamics on large geographical scales*. However, this does not solve the problem of mapping the difference between the speed of movement of persons and the rate of development of epidemics or natural disasters [36]. This is particularly important for the effectiveness of the actions taken at institutional levels.

Nevertheless, researchers have been lately discussing the following question: *How can qualitative fieldwork support big-data research?* [37] This is significant, especially as some point out that the "ease of collecting, storing, and processing" large volumes of high-resolution spatiotemporal data leads to the "fourth paradigm of science" [38].

A hybrid approach combining quantitative and qualitative data sources has been adopted in studies using the aforementioned Government Response Stringency Index which was first introduced by the Oxford COVID-19 Government Response Tracker. It codes qualitative policies into numbers and then takes the average of these specific policies such as school closure, business closure or public event cancelation.

The current availability of big data gives particular importance to algorithms that allow for the creation of geographical knowledge, referred to as algorithm-driven geographies (or algorithmic geographies) rather than data-driven geography [39]. In human mobility studies, the use of large datasets by providers such as Google, Apple or Huawei allows simultaneous observation of mobility changes [40]. This is significant, because even though the content-oriented approach to mobility research has allowed for an in-depth view of spatiotemporal changes, it did so with considerable limitations in the scope of the study.

Much of the research on mobility relates to urban studies, due to the high quality of geolocation data in urban areas and the data density associated with the number of users of mobile technologies [41–44]. Although the big data approach has already entered the mainstream in urban studies, a multidimensional look at these issues is required due to new real problems emerging in urban spaces [45]. This means that many different data sources need to be combined, which allows scientists to conduct research and solve problems in urban studies. The heterogeneity of data sources enables better spatial mapping of mobility and increases the number of contexts in which data can be analyzed [46].

This approach to showing changes in human mobility has been used in studies presenting human movement patterns in the Covid-19 context based on geotagged Twitter data [47]. The processing and visualization of these data in space, combined with the analysis of other databases, showed a significant decrease in the number of trips caused by the introduction of further constraints related to combating the pandemic. Similar observations confirm the results of studies for bike-sharing usage during the Covid-19 pandemic [48].

Our contribution to mobility studies is to show the path that has been used to integrate and validate two publicly available large datasets, shared by Google [https://www.google.com/covid19/mobility/] [49] and John Hopkins University [https://github.com/CSSEGISandData/COVID-19/tree/master/csse_covid_19_data/csse_covid_19_daily_reports] [50], which fulfill the conditions of big data. The quantitative approach, methodology and steps we have adopted in the research are universal and can be used by researchers to present any phenomena in different areas of mobility studies.

## Research methodology

### Covid-19 John Hopkins University database processing

**Fig. 1:**
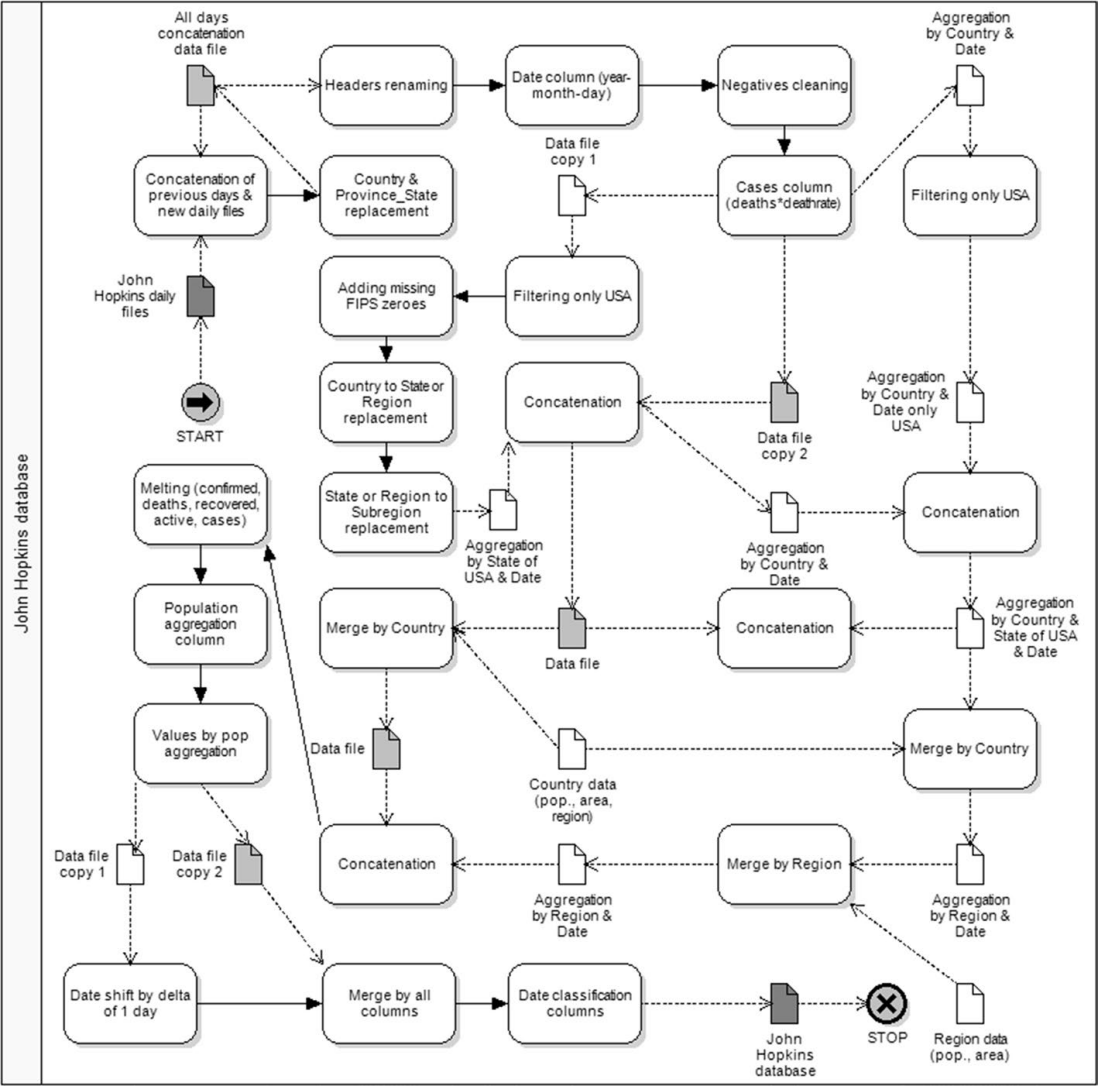
John Hopkins University data processing process

Our study uses data on Covid-19 provided by John Hopkins University. The data were processed according to the diagram shown in Fig. 1. John Hopkins University source data (file 1: f1_Covid19-Hopkins_Source.csv) are the only dataset that can still be edited in MS Excel. Although the number of rows of source data exceeds 1 million, the number of rows still falls within the limit (1,048,576 rows) of MS Excel rows.

The information concerns the history of Covid-19 and has the form of CSV files for each day of analysis. The data were retrieved as separate files from each day (338 files), cleaned and combined into a single collection with an additional column containing the date of the file from which the data originate. Even though the data contain a time classification column (date—time), we introduced another date column to track the daily variability of the data based on the name of the files from which they originate.

The variables used for the analysis refer to data containing the sum of the daily "confirmed" cases, the sum of “active” cases, the sum of “deaths” and the sum of people who have "recovered” from the disease. At the same time, for further research, we introduced another dynamic variable "cases" defined as a theoretical number of cases based on the number of deaths at a given mortality rate: cases = deaths*death_rate (initially adopting the mortality rate as 1%). The data were cleaned by replacing values in the database based on Table 1.

**Table 1:** John Hopkins University data replacing table

John Hopkins University data replacing table			
Before	After	Before	After
French Guiana	France	West Bank and Gaza	Palestine
Guadeloupe	France	Taiwan*	Taiwan
Vatican City	Holy See	Hong Kong SAR	Hong Kong
Korea, South	South Korea	Diamond Princess	Ships
Martinique	France	Congo (Brazzaville)	Congo-Brazzaville
Mayotte	France	Congo (Kinshasa)	Congo-Kinshasa
MS Zaandam	Ships	occupied Palestinian territory	Palestine
Republic of Moldova	Moldova	UK	United Kingdom
Republic of the Congo	Congo-Brazzaville	US	United States
Saint Barthelemy	France	Viet Nam	Vietnam
The Bahamas	Bahamas	Burma	Myanmar
The Gambia	Gambia	Cabo Verde	Cape Verde
Jersey	United Kingdom	Channel Islands	United Kingdom
Georgia	Georgia US	Timor-Leste	East Timor
occupied Palestinian territory	Palestine	Faroe Islands	Faroe Islands
New York City, NY	New York	New york city	New York
New York County, NY	New York	New york	New York
		Grand Princess	Ships

Changes can be divided into three groups. The first group is the conversion of classification values to the same corresponding values in all databases used in the research. For example, we treated Puerto Rico as a separate country, not part of the United States, or converted value from "US" to "United States”. We have used three databases, two publicly available and one original. The second group of changes concerned the removal of various markings of missing data. For example, we have replaced "Unassigned" or "Unknown" with an empty cell.

The third group of modifications concerns the harmonization of the attribution of Covid-19 cases to specific passenger ships, regardless of their mooring location, and excluding them from the total number of cases for particular countries. Due to the presence of location data (longitude and latitude), the missing FIPS codes for the same locations could be completed. At the same time, location data were supplemented with longitude and latitude values with the highest accuracy, defined as the largest number of characters for a given combination of a country, province, state, district or city.

We sought to limit the number of potential countries with small populations that could distort the results because of their uniqueness. On the other hand, we wanted as large a population as possible to be indicative of the results, so instead of removing territories politically dependent on a major country, we combined them, assuming that the response of individual governments to Covid-19 is consistent across the territory under the same political control. We also assumed that dependent territories, even located far away from the main territory, share a common culture.

The post-cleaning source data included a time frame between January 22, 2020 and December 24, 2020, for 199 countries (column: Country) and 667 provinces or states in these countries (column: Province_State), and 1957 unique districts or cities in these provinces or states (column: Admin2). The data show a detailed breakdown within the United States. The three above classification columns (countries, provinces, districts or cities) were translated into 4131 combinations.

The combined and initially cleaned data were further processed in the value column by converting negative active cases (“active”) into a positive number of cured cases (“recovered”). This was due to the misallocation of the number of recovered cases into the column of active cases in the processed John Hopkins University database.

To show U.S. states as the equivalents of countries, the country classification column and the province or state column have been swapped. The same was done by shifting the districts to the place of the provinces or states. Another classification column has been added to highlight the location of the shift to preserve the possibility of grouping individual states.

Subsequently, we added further classification columns with information on countries or states based on countries and regions database (file 2: f2_Country_regions.xlsx). This provided information on the two-letter designation of the country (ISO code) or state (the first two FIPS digits), the population of the country or state, and the region of the world to which the country belongs. The following were specified: Asia and the Pacific countries: "ASIA", Western Europe: “WE", the remaining countries of Europe, Africa and Asia Minor not belonging to Western Europe: "EEMEA", South America: "SAM", North and Central America: "NAM & CAM".

For countries or states, 100,000 multiplicities were calculated to two decimal places based on the population column. This allowed us to show data from the John Hopkins University database against the background of the population size of any spatial unit. On this basis, the values were converted into the population size of the country or state. Then, for the column containing values, the daily increments of all variables were calculated as differences between this day’s valued and the previous day's value. Daily increments per 100,000 inhabitants were also calculated for countries or states.

The rolling averages of 7 and 14 days were calculated for all five variables for each column: daily Covid-19 values, values per population and daily increments of both figures. For a symmetrical window size of 14 days, the value was placed next to the last value of the first half of the window. We also added the classification column to filter out the missing days of the data range end, the last 3 days for the 7-day average and 7 days for the 14-day average, respectively.

Based on the date of publication of the source files, we created time classification columns: month (number), month (text), week of the year, day count from the beginning of data for a given area (defined as: country or state—province—district or city), week count from the beginning of data for a given area, day of the year, day of the week (number), and day of the week (text).

Summing up the John Hopkins University database data processing path shown, we obtained the total database of 5,579,680 rows and 46 columns: 256,665,280 data cells (file 3: f3_Covid19_Hopkins.csv).

### Google Mobility database processing

The second base we used in the research was the Google Mobility database which was made available for research by the company Alphabet. The data for a given day include the sum of daily mobility decreases compared to base mobility divided by type of mobilities. These include: “retail & recreation areas, "groceries & pharmacies", "parks", "transit stations", "workplaces” and "residential areas". The data were cleaned by replacing values in the database based on Table 2.

**Table 2:** Google Mobility data replacing table

Google Mobility data replacing table			
Before	After	Before	After
Côte d'Ivoire	Cote d'Ivoire	Réunion	Reunion
Myanmar (Burma)	Myanmar	Georgia	Georgia US

Google Mobility database uses differentially-private analysis which adds noise to results to anonymize the location data of individual mobile phones. This ensures that the data do not show individual activity patterns while preserving the usefulness of the data at scale. These reports are intended to show the changes that have taken place around the world, following the introduction of restrictions regarding the Covid-19 pandemic.

At the same time, for further research, we have introduced a variable "Combined" defined as the resultant of the weighted average of some of the mobility types we consider key, with the following weights in order of importance: 0.4 for "retail & recreation areas", 0.3 for "transit stations", 0.2 for "workplaces", 0.1 for "groceries & pharmacies" and 0 for "residential areas" and "parks". The adopted "Combined" weights were an educated guess based on an analysis of the positive-to-negative correlations between different types of activity resulting from a preliminary analysis made on the first 179 days of data from the Google Mobility database. The above weights have been adopted a priori, but a more accurate determination of the optimal weights is possible on the basis of an analysis of the different options. We searched for the maximum square value of Pearson's correlations of the highest daily mobility decreases offset in time towards the daily increments in new Covid-19 cases detected.

Although the Python version 3 programming environment was used to process the source data of both databases, we used the CORREL(X,Y) function of MS Excel to calculate the correlation of the resulting data.

As with the John Hopkins University database, the data were cleaned by swapping 4 values to convert classification values to the same corresponding values from the corresponding databases.

The post-cleaning source data included a time frame between February 15, 2020 and December 11, 2020, for 135 countries and 1,860 provinces or states in these countries, and 9914 unique districts or cities in these provinces or states and additional 65 metropolitan areas. The three above classification columns translated into 13,269 combinations. Data in the wide format accounted for 3,468,548 rows, which, with 7 variables (including the dynamically calculated “Combined”), translated into 24,279,836 rows in the long format.

The data end on December 11, because after this period in some countries (e.g. USA or the UK) a mass SARS-Cov-2 vaccination campaign was launched, which is why activity data after this period will be characterized by systemic disorder. Vaccinated individuals will be able to be active with minimal impact on the number of Covid-19 cases. In addition, universal vaccination programs are large-scale logistic operations, carried out mainly by medical staff using strict personal protection measures, which increases the level of activity of societies with minimal impact on the number of Covid-19 cases.

To show U.S. states as the equivalents of countries, the country classification column and the province or state column have been swapped. The same was done by shifting the districts to the place of the provinces or states. Another classification column has been added to highlight the location of the shift to preserve the possibility of grouping individual states.

For the column containing values, the daily increments were calculated as differences between this day’s value and the previous day's value. In addition, the data from the Google Mobility database were offset in time in relation to the John Hopkins University database. The offset level can be changed dynamically. For the analysis of the relationship between mobility and the spread of the pandemic, the original time data were also preserved.

The rolling averages of 7 and 14 days were calculated for all seven variables for each column: daily mobility values, offset values and daily increments of both figures. For a symmetrical window size of 14 days, the value was placed next to the last value of the first half of the window. As with the John Hopkins University Covid-19 database, we also added the classification column to filter out the missing days of the data range end (3 days for the 7-day average and 7 days for the 14-day average, respectively).

**Fig. 2:**
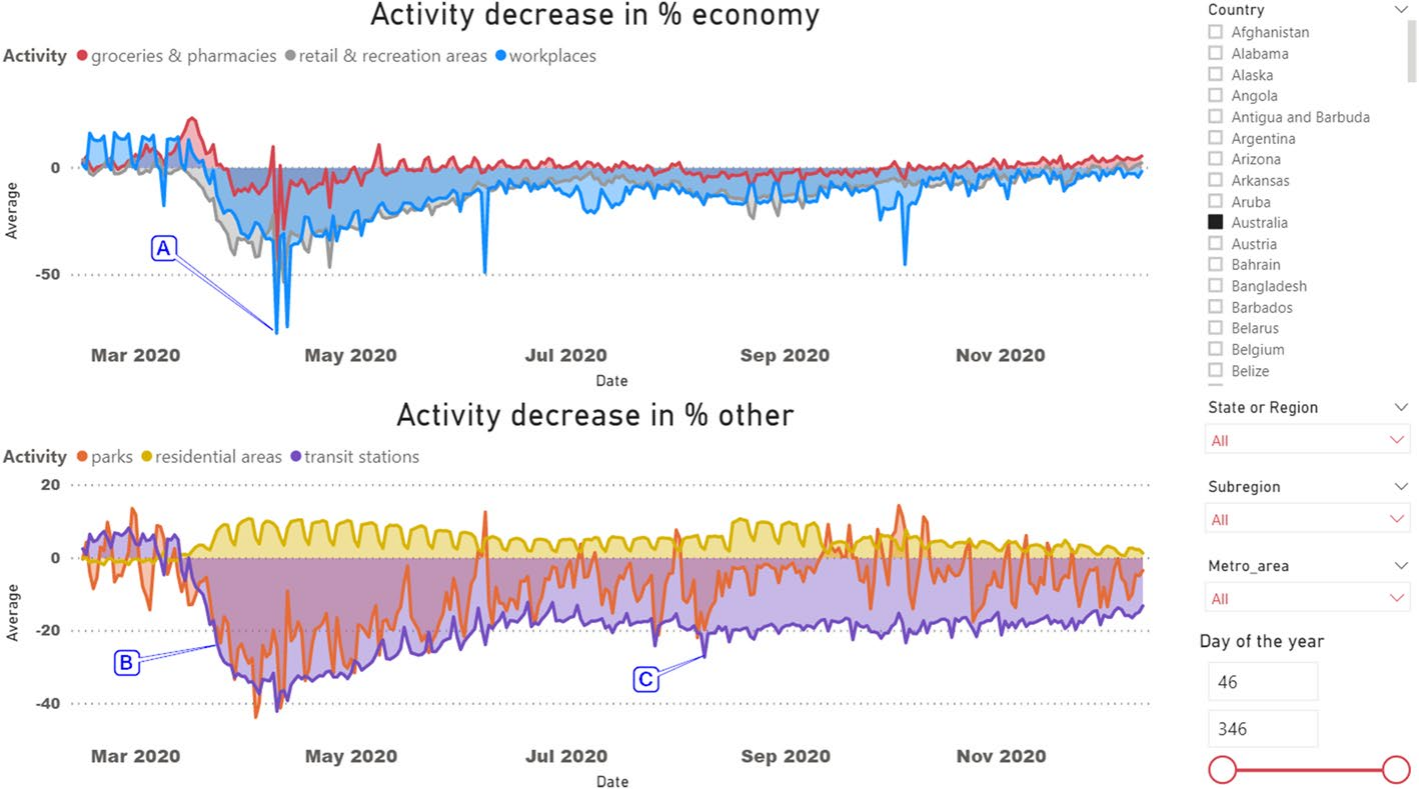
Google Mobility for Australia visualized in MS PowerBI dashboard

The data operations performed gave a total base size of 24,279,836 rows and 33 columns: 801,234,588 data cells. The above mobility database has been loaded into a business intelligence type software (MS PowerBI dashboard shown in Fig. 2) for more convenient data visualization (file 4: f4_Google_Mobility.pbix).

We chose Australia because of the representativeness of the characterization of activity changes allowing us to illustrate phenomena frequently occurring during the lockdown, which makes it impossible to simply take the lockdown center as if it were a point of minimal activity throughout the year. However, we could not show the average because of the different lockdown time ranges for each country.

Our assumptions about the location of the lockdown center are debatable and we hope that other researchers will use the data we collected to better locate lockdowns using other algorithms. Our preference to use the data without comparing them to the dates of legislation sanctioning lockdowns results from the fact that in some countries regulations have been repeatedly changed and updated. The unique case of Sweden further illustrates that lockdown can be introduced as a voluntary recommendation made at a press conference rather than a legal obligation. Actual population activity data derived directly from the data take precedence over legal considerations that only give an idea of population activity recommendations, not their implementation.

In Appendix A, Table 6, we presented the lockdown taxonomy based on mobility changes. In addition, Figs. 10, 11, 12, 13, 14, 15, 16, 17, 18, 19, 20, 21, 22, 23, 24, 25, 26, 27, 28, 29, 30 contain visualizations for the countries we included in the taxonomy.

## Results

### Combining data into the Mobility-Covid-19 database

**Fig. 3:**
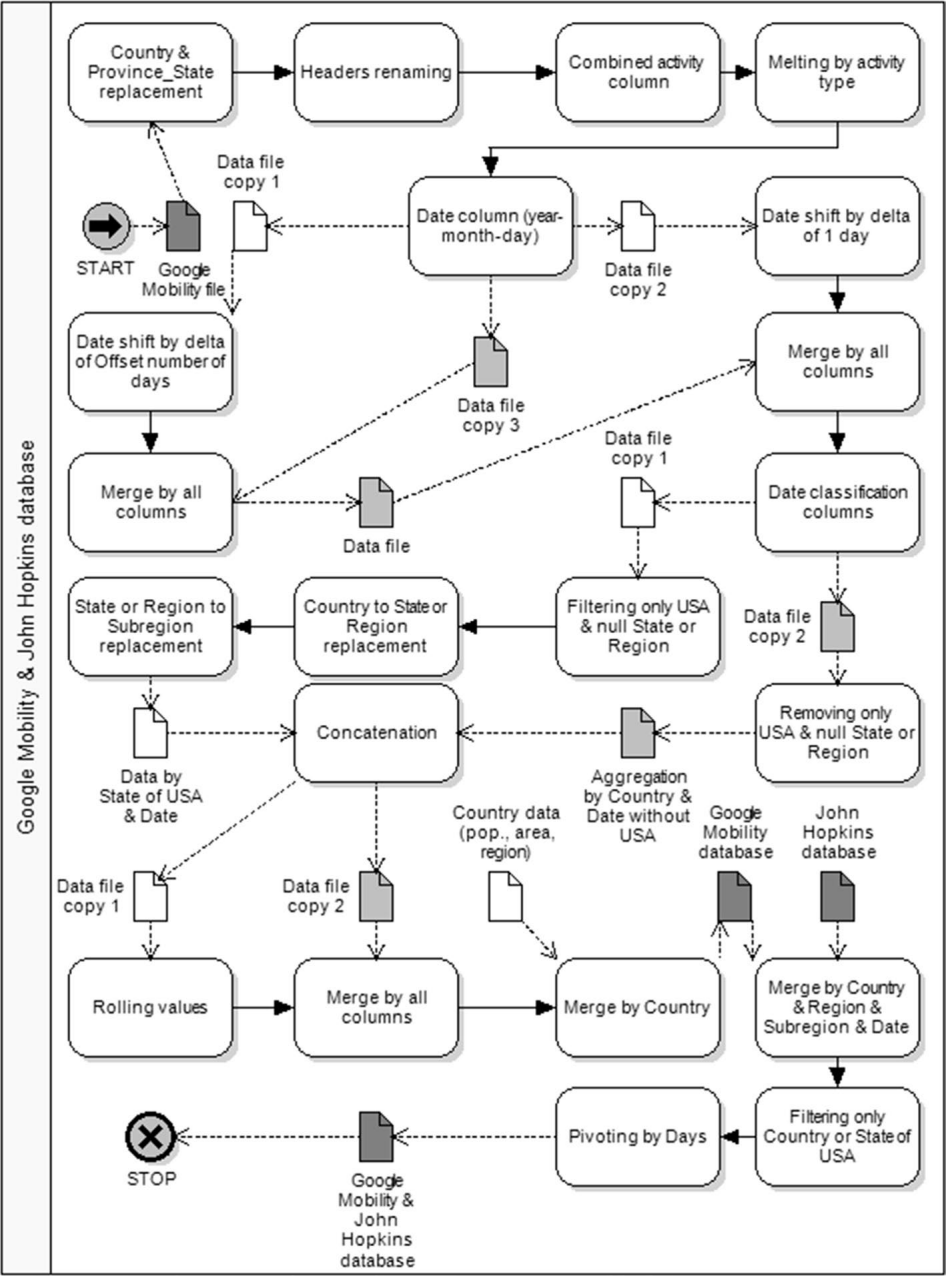
Google Mobility and John Hopkins University data processing process

The combination of both databases, the John Hopkins University Covid-19 database and Google Mobility database, takes place at the level of the larger base, namely, the Google Mobility base with 13,269 combinations of spatial markers. The data were processed according to the diagram shown in Fig. 3. They refer to countries, provinces or states in these countries and unique districts or cities. The Covid-19 database of John Hopkins University contains 4,131 combinations. This research approach stems from the adopted assumption that human mobility has impact on the spread of the pandemic.

One variable can be linked to the Google Mobility database each time, e.g. the number of daily “confirmed” cases or the number of “deaths” from the Covid-19 database (5,579,680 rows), corresponding to the same combinations and time series days.

In our study, we limited the Mobility-Covid-19 result base to overlapping combinations of countries and individual U.S. states (185 cases). This is because these were the only combinations for which we had population size data that could be used to weigh the correlations between the values from both bases. The second reason for this intervention was the need to limit the analysis to large population groups. This eliminates the disruption of daily increments caused by local outbreaks of the disease. This has reduced their impact on statistics in a given group in situations of rapidly increasing incidence in places where people are forced to reside in confined spaces, such as locked facilities or nursing homes.

From the combined Mobility-Covid-19 database, the following columns were exported: mobility type, country or state, country or U.S. state it belongs to, population size, region of the world it belongs to, offset mobility value for the rolling average, value of daily case increments for the rolling average, and day count from the start date. To show the regional diversity of lockdown, the study highlighted 5 geographical regions.

For the 7-day and 14-day rolling averages, a 50-fold database data export operation was performed for different values of the mobility time offset intervals, with offset values from 1 to 25 days (1 export for each offset separately for 7-day and 14-day rolling averages—50 in total).

The operation was then repeated for a subgroup of the first 150 days and subsequent 151 days, which translated into 150 exports. In addition to the combined data set, we used the first and second half as they include different waves of the virus propagation—spring and autumn wave. The argument for analyzing the additional two databases in the form of the first and second half of the dataset is that it describes different variations of the virus. The first SARS-CoV-2 variant that triggered the spring wave (in line with evolutionary pressures preferring lower mortality and higher contagiousness) in the autumn already had an admixture of new virus varieties with a higher R0 (number of people infected by one infectious individual). Moreover, a virus with a lower mortality rate may have a different incubation period. By comparing the values of the offsets with the highest correlation from the first and second waves, we can determine whether the incubation period is lengthened or shortened. Previous studies have assumed that the average incubation period for this virus is 7–14 days and the time required for the recovery or death to occur is about 10 days based on several factors, such as weather, environmental conditions, average ages of people and natural immunity against viral diseases [51].

An additional argument for separating the base was the fact that the spring wave featured a greater degree of reduction of activity. The maximums from the combined base mostly include maximum reductions of spring activity, which largely disregards the characteristics of the autumn lockdowns.

In this way, for further analysis, we obtained three result datasets for “confirmed” cases. First dataset contains all 301 days of mobility data combined with the number of confirmed cases. These data translated into 63,768 rows and 610 columns: 38,898,480 data cells. Additionally, we created separate dataset for the first 150 days of mobility data which translated into 63,768 rows and 308 columns: 19,640,544 data cells. Eventually, we created a separate set for the last 151 days of mobility data which translated into 62,726 rows and 310 columns: 19,445,060 data cells (file 5: f5_Results_25_Confirmed.xlsx). These three datasets are mirrored by another three for “deaths” but those results were not presented because maximum offset of 25 days did not cover the maximum correlation between offset mobility data and Covid-19 data (file 6: f6_Results_25_Deaths.xlsx).

### Human mobility impact on Covid-19 transmission

Based on the created Mobility-Covid-19 database, we analyzed the correlations between the decrease of mobility for all variables according to the Google Mobility database and the increment of daily cases for the variable "confirmed”, according to the Covid-19 database. We also determined the optimal mobility time offset interval for the Covid-19 database.

During the database analysis, frequent asymmetric situations were detected against the maximum values on the global chart of daily increments of Covid-19 cases. This occurred when the initial dynamic increase in the number of daily new cases (after exceeding the maximum) was followed by a slow decline in the number of cases, while maintaining a steady level of low population mobility. Moreover, it is likely that with the loosening of restrictions, the increasing fatigue of the society and a high level of mobility decline, we can observe a slow return to a higher degree of mobility in many cases. At the same time, we are observing a further decline in daily Covid-19 increments, resulting in a negative correlation between population mobility levels and daily increments in new cases after exceeding the maximum.

Theoretically, the maximum lockdown point should be the point with the lowest activity—the global minimum for a given type of activity (Fig. 2 point: A). However, as soon as the level of activity reduction is reached, at which (after the incubation period) the number of cases will decrease, the upward trend will reverse. Free propagation of lockdown information in society is the reason why lockdown is never instantaneous and is characterized by an exponential increase in the daily percentage decrease in activity, reaching its maximum and an exponential percentage decrease in activity decline (Fig. 2). Moreover, sometimes the maximum point is reached very steadily (Fig. 2 point: C) and only after many weeks, the activity reaches the minimum. This is why the growth inflection point—the decrease in the number of cases—can be exceeded long before the activity reaches the global minimum.

For this reason we decided to define the maximum lockdown as the largest decrease in activity defined by the largest daily increment in activity drop percentage. In the charts in the PowerBI dashboard, this will be the largest angle of decrease of activity (Fig. 2 point: B). As a result, the lockdown window is referred to the date on which such a maximum percentage decrease occurs by subtracting and adding a certain number of days to the correlation analysis.

Taking this into account, we have decided to study the global maximums of the decline in mobility. They represent the highest daily mobility increments expressed as a percentage across the time series against daily increments in Covid-19 cases. In other words, the lockdown’s impact was investigated on the assumption that during its course (usually half through), the maximum daily decrease of population mobility occurred (calculated as a percentage compared to the average level of mobility in a given area).

The time series analyzed was limited to the time window set by the global maximum daily population mobility declines in each type of mobility and the number of analyzed days. In our analysis, we examined 13 different window sizes (from 5 to 29 days with 2 day increment) associated with different numbers of days of lockdown analysis in terms of the average square of correlation. The maximum occurred half way through the interval.

Then we selected the window size which translated into the maximum values of the average square of correlation of all of over 63,768 cases studied in 13 different window size variants. The number of cases results from all combinations of mobility types, countries and states in two versions of data smoothing, with a 7-day and 14-day rolling average, tested in 25 variants of offset in relation to the John Hopkins University database.

**Fig. 4:**
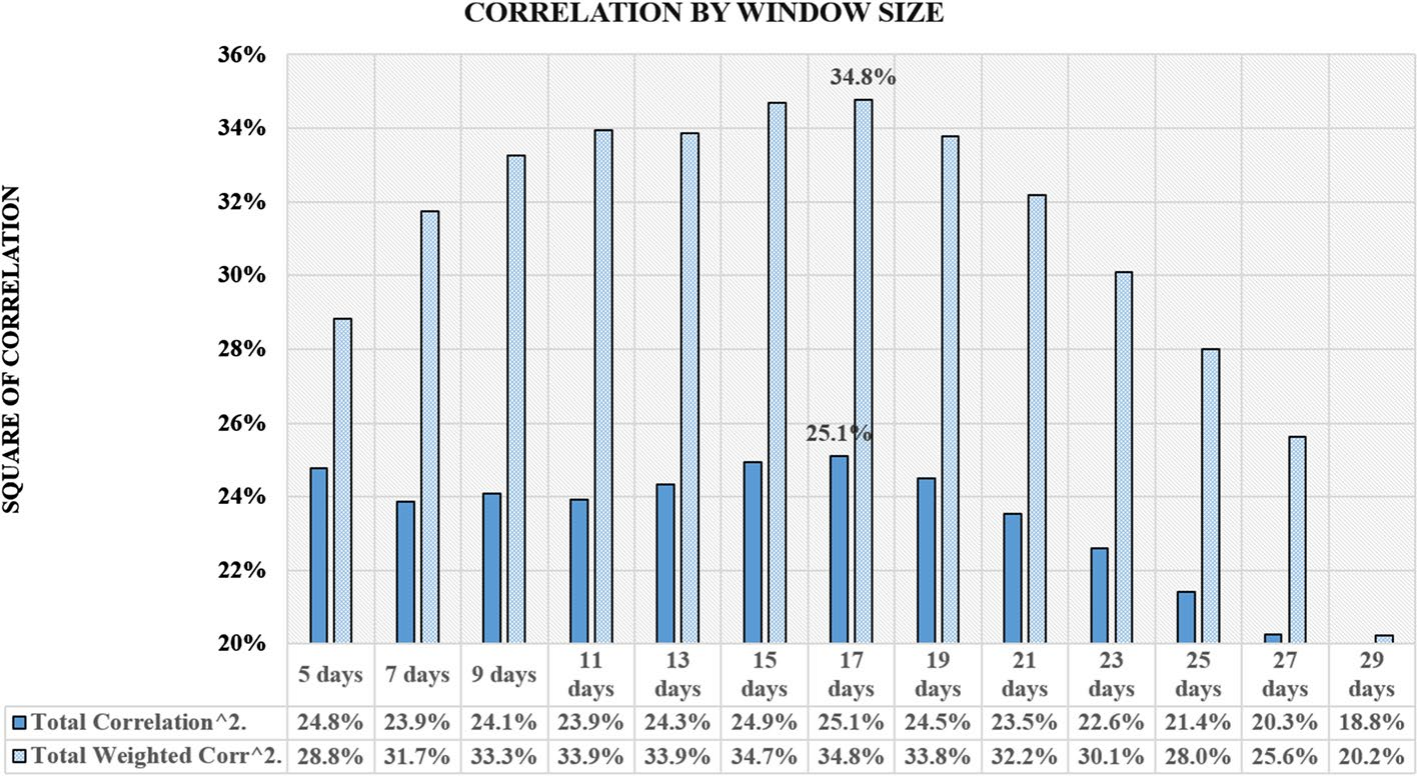
Correlation by window size for all data

The analysis of the results from the full scope of 301 days of data is shown in the graph (Fig. 4) as the average of the square of correlation and as the weighted average of the population size of each country or state. The promising window size is 17 days.

**Fig. 5:**
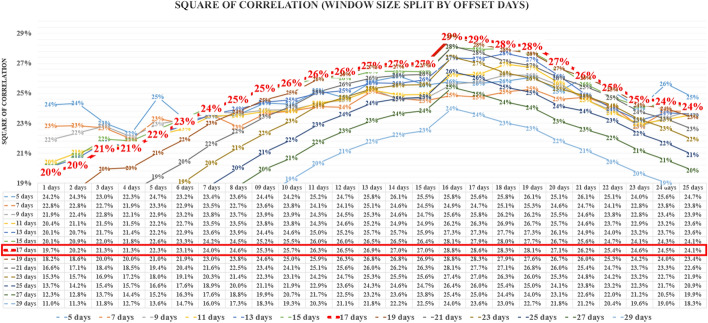
Square of correlation by window size split by offset days

The level of the average square of correlation of the selected test window for 63,768 cases was verified over the entire range of possible intervals of offsetting data on population mobility decline relative to the Covid-19 new cases, as shown in the graph (Fig. 5). The most promising window size: 17 days, is highlighted in larger bolded font.

Based on the results, the 17-day window size was selected. After choosing the size of the window, the correlation was measured on a full base of 301 days until December 11, as well as on the first half of the base of the first 150 days covering the spring wave of Covid-19 and the second half of the base of the subsequent 151 days covering the autumn wave of Covid-19.

**Fig. 6:**
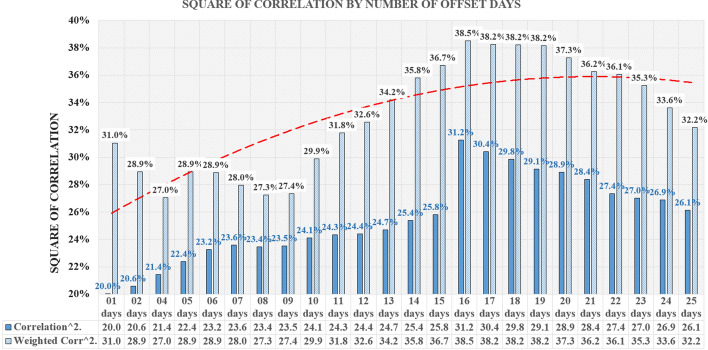
Square of correlation by number of offset days—all 301 days of analysis

For each of over 63,768 cases, the average square of correlation was then analyzed to determine the number of days of the time offset of the Google Mobility database in relation to the John Hopkins University database. Figure 6 represents the average square of correlation along with the trendline (Polynomial: order 2) for the first data collection which included all 301 days of analysis. In the chart, we showed the average square of correlation and the average weighted by the population size. The trendline refers to the weighted average.

**Fig. 7:**
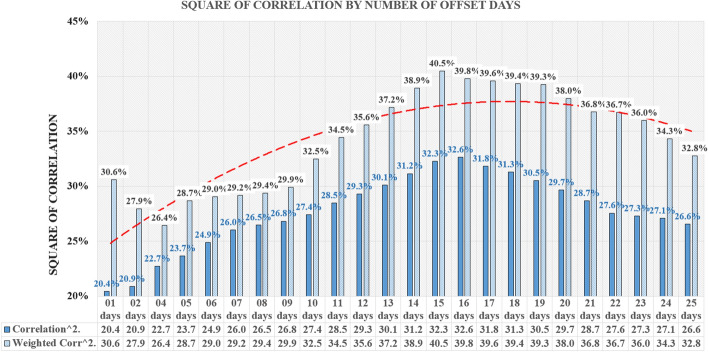
Square of correlation by number of offset days—first 150 days of analysis

Figure 7 represents the average square of correlation along with the trendline (Polynomial: order 2) for the second data collection which included the first 150 days of analysis—the spring wave.

**Fig. 8:**
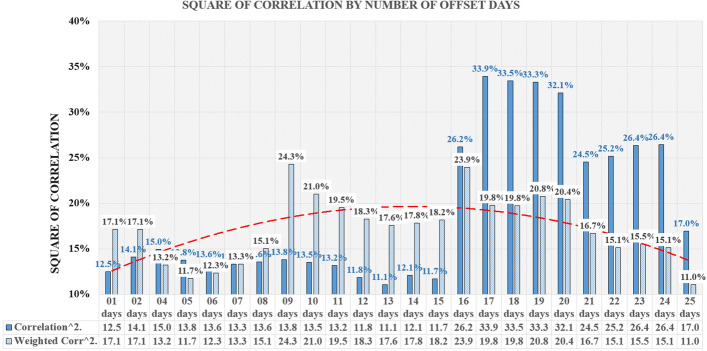
Square of correlation by number of offset days—last 151 days of analysis

Figure 8 represents the average square of correlation along with the trendline (Polynomial: order 2) for the third data collection which included the last 151 days of analysis—the autumn wave.

**Fig. 9:**
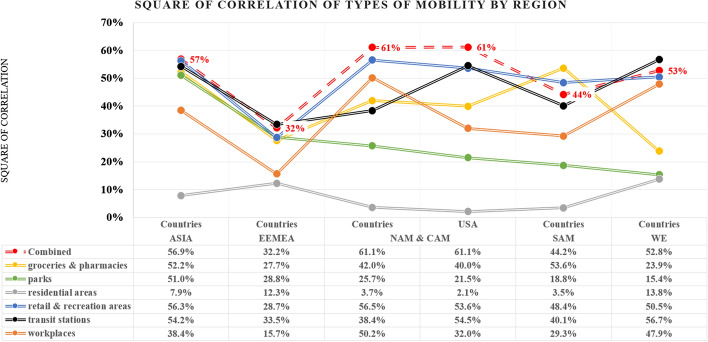
Square of correlation with a 16-day offset of mobility types by region

The overall trend determines the highest value of the average square of correlation with a 16-day offset. Based on all 301 days of analysis, Fig. 9 represents the average square of correlation with a 16-day offset of mobility types by countries of a specific region.

With the exception of the average for South American countries (group “SAM”), further studies testing different weights for individual mobility factors may answer the question of whether South American countries have a different relationship between activity declines and daily increments of Covid-19 cases, or whether it is a question of suboptimal pre-determination of weights in the studies conducted.

At the same time, the above summary occasionally indicates serious differences between the values of square of correlation for different regions of the world. This may be due to differences in terms of the wealth of societies and levels of social communication, as the differences between the best-researched “WE” and “USA” regions are minor. This can be explained by the similar level of human mobility of both societies and cultural affinity. In addition to the above observations, we can distinguish relatively constant correlation characteristics for a given type of mobility, although further research is required to explain the major differences in individual regions.

Another observation on the average levels of the square of correlation is to examine which smoothing degree affects the level of the average square of correlation. Based on all 301 days of analysis, Table 3 shows the results of calculations by the level of data smoothing.

**Table 3:** Square of correlation by rolling window of mobility type by region

Square of correlation by rolling window of mobility type by region table								
Rolling	Activity	Countries	Countries	Countries	USA	Countries	Countries	Total Weighted Corr^2
		ASIA	EEMEA	NAM & CAM	NAM & CAM	SAM	WE	
Mean of 7 days	Combined	24%	19%	13%	4%	12%	24%	15%
Mean of 7 days	Groceries & pharmacies	17%	14%	19%	28%	19%	13%	19%
Mean of 7 days	Parks	15%	15%	8%	15%	16%	15%	14%
Mean of 7 days	Residential areas	9%	14%	6%	5%	2%	9%	9%
Mean of 7 days	Retail & recreation areas	19%	18%	9%	5%	18%	28%	15%
Mean of 7 days	Transit stations	21%	19%	14%	23%	21%	33%	22%
Mean of 7 days	Workplaces	15%	11%	11%	6%	20%	16%	11%
Mean of 7 days Total		17%	16%	11%	12%	15%	20%	15%
Mean of 14 days	Combined	33%	32%	28%	42%	46%	43%	36%
Mean of 14 days	Groceries & pharmacies	32%	23%	26%	32%	29%	23%	27%
Mean of 14 days	Parks	21%	25%	28%	22%	32%	13%	23%
Mean of 14 days	Residential areas	12%	11%	13%	3%	19%	8%	9%
Mean of 14 days	Retail & recreation areas	35%	29%	37%	38%	45%	40%	35%
Mean of 14 days	Transit stations	26%	30%	30%	46%	44%	43%	36%
Mean of 14 days	Workplaces	22%	19%	28%	15%	25%	38%	22%
Mean of 14 days Total		26%	24%	27%	28%	34%	30%	27%
Grand Total		22%	20%	19%	20%	25%	25%	21%
Difference: (Mean14 -Mean 7)/Mean 7		51%	56%	137%	132%	123%	50%	81%

The level of results improvement (the difference between the results in relation to the 7-day average result) for all types of mobility varies from 51 to 170% depending on the type of average (normal or weighted) and region. Even though only two levels of smoothing have been studied, the above results suggest that the commonly used level of smoothing in the Covid-19 studies [52] in the form of a 7-day rolling average may not be optimal and should be replaced by a higher value.

The main results of our study based on all 301 days of analysis concern the flexibility of individual activities and their combination against the number of Covid-19 cases based on an analysis of the ratio of positive to negative correlations. For activity in places of residence, "residential areas", negative correlations (101 to 69) prevail. This is the only type of activity with a negative correlation. This could be expected, as the reduction in the number of Covid-19 cases should depend on increased activity in places of residence. Other activities have a positive relationship between reducing activity and reducing the number of Covid-19 cases (result base extract for a 16-day time offset, only with 14-day smoothing) (Table 4).

**Table 4:** Positive to negative ratios by type of activity

Positive to negative ratios by type of activity table			
Type of activity	No. of correlations		Ratio of ( +) to (−)
	Positive	Negative	
Groceries & pharmacies	125	53	236%
Parks	119	58	205%
Residential areas	69	101	68%
Retail & recreation areas	147	31	474%
Transit stations	142	35	406%
Workplaces	132	45	293%
Combined	141	33	427%
Total	875	356	246%

The prevalence of positive correlation is the smallest (except “residential areas”) in green areas, "parks", with a positive to negative ratio of 205%. The question "*Can the differences in the proportion between positive and negative correlations be used to better determine weights for "Combined" resultant activity (which has reached a proportion of 427%, however, “retail & recreation areas” are still higher with a proportion of 474%)?*" requires further research focused on correlation distributions, and not only positive–negative analysis.

## Discussion

The study analyses location features of the impact of human mobility on the spread of Covid-19 at a global level.

Lockdown in our study is defined as the highest percentage of activity reduction over a time series extended by an appropriate number of preceding days and the same number of days following the maximum reduction date. An adequate number of days showing the highest level of correlation was indicated after searching a wide range of possibilities. The level of data smoothing in the Covid-19 and Mobility databases, on the other hand, included a 7- and 14-day moving average. This has allowed us to exclude frequent indications of a single day with a large reduction in activity as a temporary lockdown center. Therefore, due to the inclusion of the actual strongest reductions in population activity, the study refers to all possible types of lockdown. The global approach to lockdown eliminates the impact of political considerations and ways of communication or lack thereof (as in Sweden) in individual countries or states of the USA.

Our research has revealed that it is more relevant to look for a relationship between human mobility and Covid-19 transmission adopting a 14-day moving average. This observation is important, because much of the previous research into the impact of mobility reduction on Covid-19 transmission was based on smoothing data streams by using the 7-days rolling averaged mobility, leading to a significant difference in correlation analysis results [53, 54]. This also applies to the sample size, which for the mentioned studies was 52 countries.

The impact of population activity limitation on the reduction of new cases of the Covid-19 virus has been demonstrated on the example of a large number of countries, including individually studied U.S. states. However, it does not apply to residential areas. The positive correlation remains high regardless of the level of wealth of the countries and the cultural characteristics of a given region of the world.

The time range of our study includes changes in daily activity and the number of Covid-19 cases in the first half of the pandemic, from January 22 to December 11, 2020. It includes the occurrence of the first wave of Covid-19 for most countries of the world. Furthermore, updating the activity data and the number of Covid-19 cases for the following months may no longer translate into significant changes in the location of the maximum activity reduction for the countries surveyed. The rate of reduction in population activity may not be as rapid in the future as it was in the case of the emotional response of the population to the first wave. The evolutionary pressure exerted on the virus causes the effect over time in the form of lower mortality while increasing the contagiousness of the virus, hence future reductions of activities. They are likely to be milder due to the lower risk for individuals, which will translate into a subjective sense of lesser danger. However, the risk will not necessarily be lower for the society as a whole due to the possible increase in the number of contagions.

The study can be further developed by adding countries (not just entire geographical regions) on the basis of various additional criteria e.g. geographical, social or medical. Due to the possibility of cross-comparison of regions with similar levels of funding and efficiency of the health service (e.g. the USA and the rich countries of Europe), Western Europe (WE) has been separated from the larger region of Europe. The remaining countries have been included in the East EMEA (Europe, the Middle East and Africa) group, covering Russia, Ukraine, Belarus, Romania, Greece, Serbia, Bulgaria, Croatia, Moldova, Bosnia and Herzegovina, Georgia, Albania, Slovenia, Malta and Kosovo.

The accepted scope of research made it possible to determine the overall relationships in the division by regions, countries, and, in the case of the United States, also states.

The research demonstrates the impact of the Google Mobility database on the John Hopkins University base at the right time offset. The master database is the Google Mobility base in which we have included the number of Covid-19 cases. We have proven that a certain offset in days for mobility data correlates highly with Covid-19 transmission data. Our data also confirm that certain types of economic activity are more correlated than others. This proves not only that lockdowns are suppressing diffusion of Covid-19. Those results suggest that a selective lockdown strategy focused only on activities with the highest correlation could be more beneficial than a total lockdown strategy. This conclusion stands in contrast to the strategy adopted for dealing with the epidemic in Sweden and other countries where no lockdown has been introduced. Our results strongly suggest that retail & recreation areas type of activity has the highest contribution to Covid-19 transmission. Additionally, the most economically productive type of activity in workplaces is not the highest in the correlation ranking (Table 4) and it is far below retail & recreation areas activity. Because of that, we suggest that workplaces lockdown has the lowest Covid-19 suppression benefit to economical cost ratio so it should be applied as an action of last resort.

We have proven that the correlation coefficient is negative for home activity (“residential areas”) and positive in all other ones. With the manually defined "Combined" mobility resultant confirmed by a very high correlation. Based on the results obtained (Table 4), the following types of mobility are of utmost importance in the reduction of the Covid-19 spread, respectively: “retail and recreation areas”, “transit stations”, “workplaces” (surprisingly in the third place), “groceries and pharmacies” and “parks”. This proposal is particularly significant for government agencies which decide to impose mobility restrictions under lockdown policies. It identifies critical areas for prevention, monitoring and limitation of human mobility.

## Conclusion

Big data analytics, and in particular the use of multiple databases, including copyright, requires a clear and precise determination of the links between the data sources being processed. We see the need to document the next steps and stages in the studies so that they can be reproduced and repeated by researchers in the future. Our contribution is to present a framework for the integration of databases taking into account the topicality of the coronavirus pandemic issue.

The analysis covered all the resultants of both the Google Mobility and John Hopkins University databases, including an expanded range of common parts to include individual U.S. states. Extreme data were not filtered or adjusted to averages in the value range, so the authors avoided multiple sampling errors that could lead to biased conclusions. The results presented are derived from the processing of raw data, as the only source data that have only been filtered are those relating to entire countries or states of the USA and have not been subjected to any statistical processing prior to the correlation analysis. Analyzing more than 180 correlations and applying aggregations to large populations (entire countries or U.S. states) allowed us to obtain statistically significant results.

Studies have shown that smoothing greater than 7 days gives better results in case of a correlation between the percentage value of daily activity reduction with reference to the baseline and daily increments in new confirmed cases of Covid-19 during lockdowns. The highest correlation coefficient values were obtained for the 14-day smoothing level. The biggest differences reached 137% compared to the 7-day smoothing level. We suggest increasing the smoothing level to over 7 days, which is often used in research on Covid-19.

We have proven the link between the two bases measured by correlation force. The effect of the time offset of mobility change on the number of Covid-19 cases is practical in the current analysis of the effects of epidemic restrictions and whether they are sufficient or should be extended. An in-depth analysis of how many days should elapse to see the reduction in the number of cases is required to confirm the effectiveness of preventive actions. At the same time, first, through the percentage of positive correlations, we indirectly show which types of activity most strongly translate into the reduction of the number of cases, so that governments of countries know what activities should be focused on first (1: “retail and recreation areas”, 2: “transit stations”, 3: “workplaces”).

We have also calculated by how many days the Covid-19 base should be offset (16 days) so that this link would be the strongest for percentage declines in activity levels. Then, in the view of the fact that for the Covid-19 PCR diagnostic tests (real-time reverse transcription-PCR), the results are sometimes given a few days after the swab [55], our results fall within the incubation period of the Covid-19 virus, for which the median of the first symptoms occurred after 5.2 days, and 95% at 12.5 days [56].

Our research is limited. Out of the 185 cases analyzed, 51 are from the US, resulting in a large tendency towards the over-representation of US data. At the same time, this does not cause a distortion of the results due to the large population in each state and the fact that a similar number of tests have been performed in individual countries. The second limitation is not including China in the studies, for which mobility data are not available in the Google database. Thanks to this methodology, we have strengthened analyses and inferences through more samples in the form of spatial units.

Our future research will focus on the analysis of the offset with the highest correlation level for fatal cases (“deaths”), taking into account the range of shifts from 1 to a minimum of 60 days. In addition, regions of other countries with a high number of Covid-19 cases and a large population should be added to the analysis of those identified as the most statistically significant due to the large population.

## Figures and Tables

**Fig. 10:**
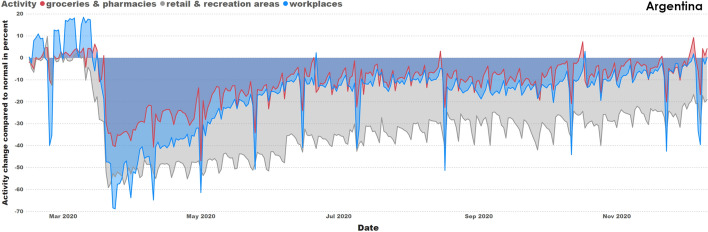
Cyclic type lockdown

**Fig. 11:**
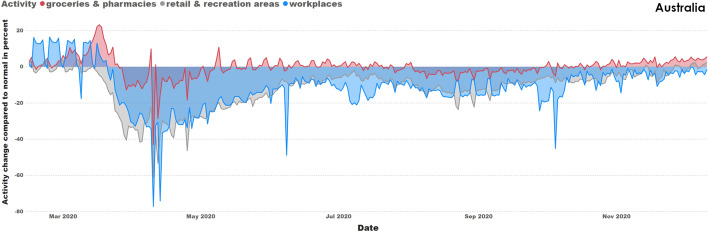
(Several) Distinct type lockdown

**Fig. 12:**
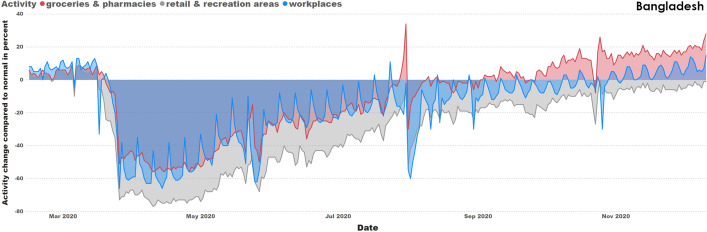
Trended gradual return to full activity

**Fig. 13:**
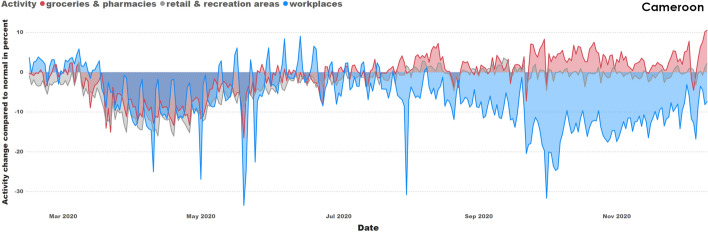
Main lockdown followed by selective workplaces reduction

**Fig. 14:**
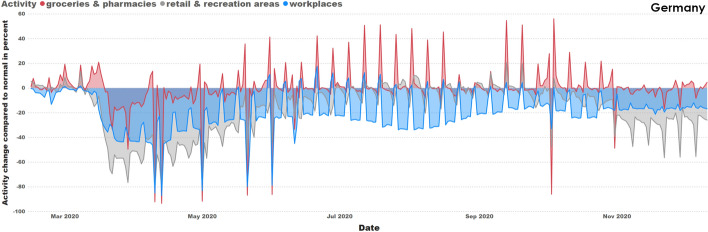
Minimal but steady reduction of activity

**Fig. 15:**
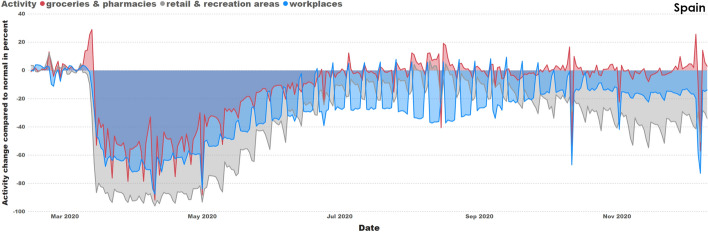
Minimal but steady reduction of activity

**Fig. 16:**
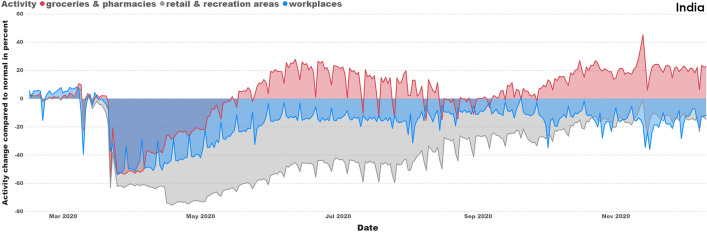
Retail and Recreation type lockdown

**Fig. 17:**
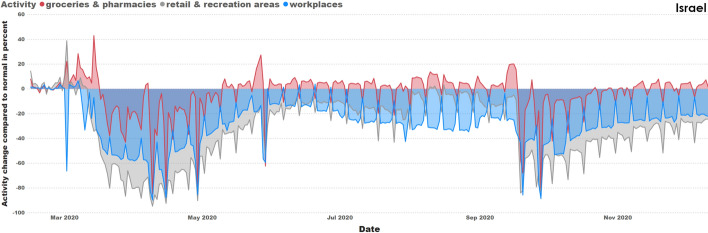
Two main with additional short duration drop in activity

**Fig. 18:**
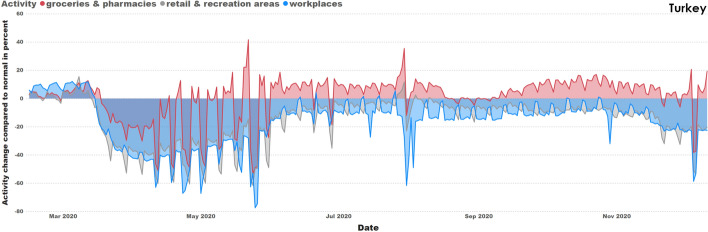
Two main with additional short duration drop in activity

**Fig. 19:**
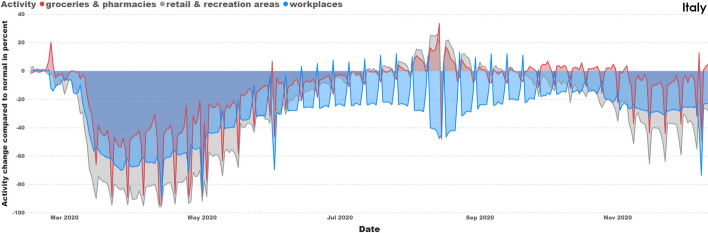
Two main with summer work activity decrease

**Fig. 20:**
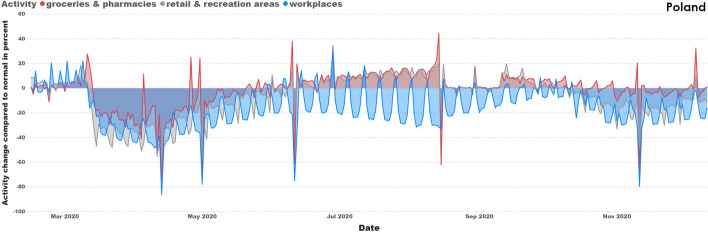
Two main with summer work activity decrease

**Fig. 21:**
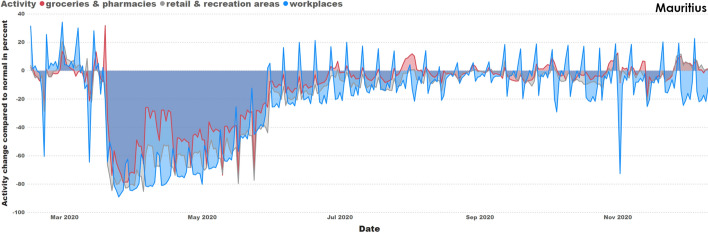
Single intense lockdown

**Fig. 22:**
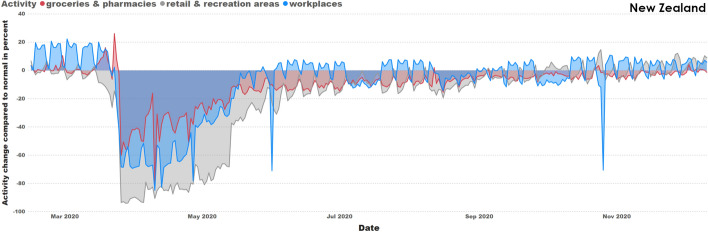
Single intense lockdown

**Fig. 23:**
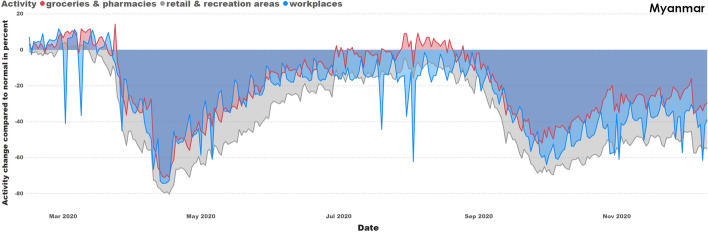
Two intense lockdowns

**Fig. 24:**
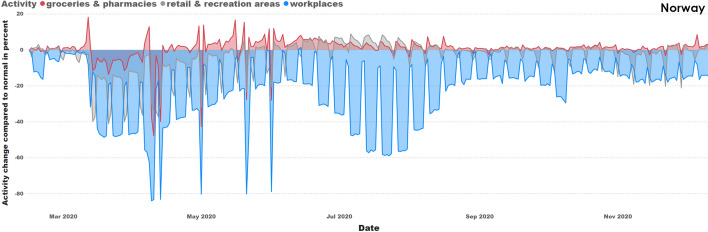
Summer time lockdown

**Fig. 25:**
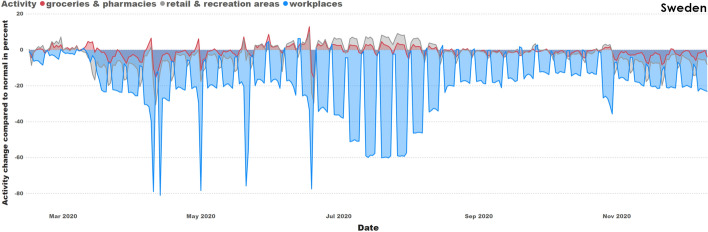
Summer time lockdown

**Fig. 26:**
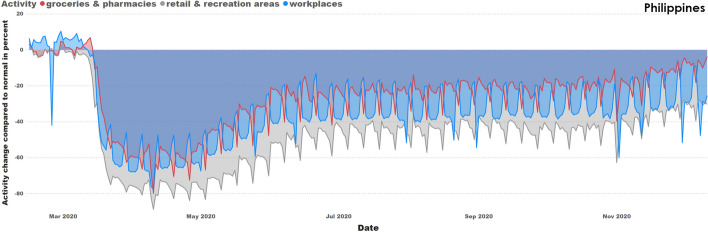
Full year intense lockdown

**Fig. 27:**
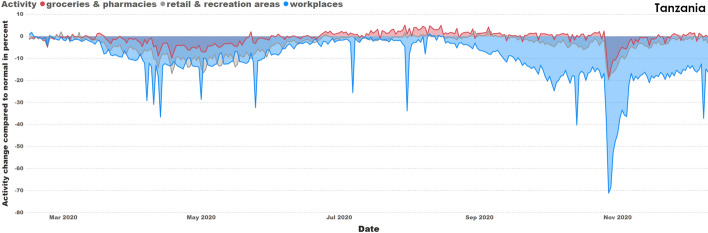
Second half of the year lockdown

**Fig. 28:**
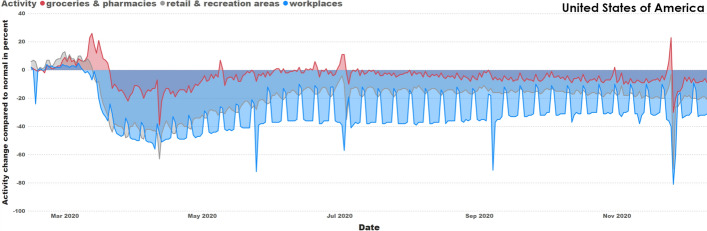
Full year lockdown with medium intensity

**Fig. 29:**
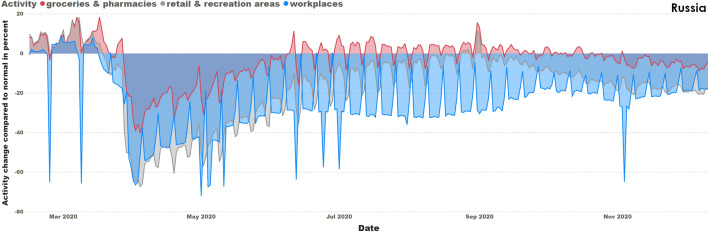
Full year lockdown with medium intensity

**Fig. 30:**
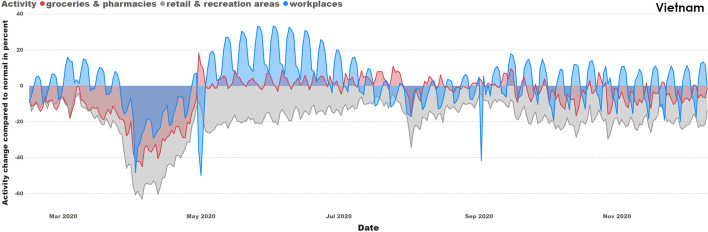
Medium intense lockdown with unusual workplaces activity increase

## Data Availability

All of data source is available in attached additional files.

## Appendix A

See Tables 5 and 6

**Table 5:** Countries and States that have been included in the studies

ASIA	EEMEA		NAM & CAM		SAM	WE
Countries	Countries	Countries	Countries	U.S. States	Countries	Countries
Afghanistan Australia Bangladesh Cambodia Fiji Hong Kong India Indonesia Japan Laos Malaysia Mongolia Myanmar Nepal New Zealand Pakistan Papua New Guinea Philippines Singapore South Korea Sri Lanka Taiwan Tajikistan Thailand Vietnam	Angola Bahrain Belarus Benin Bosnia and Herzegovina Botswana Bulgaria Burkina Faso Cameroon Cote d'Ivoire Croatia Egypt Gabon Georgia Ghana Greece Guinea-Bissau Iraq Israel Jordan Kazakhstan Kenya Kuwait Kyrgyzstan Lebanon Libya Mali Malta	Mauritius Moldova Morocco Mozambique Namibia Niger Nigeria North Macedonia Oman Qatar Reunion Romania Russia Rwanda Saudi Arabia Senegal Serbia Slovenia South Africa Tanzania Togo Turkey Uganda Ukraine United Arab Emirates Yemen Zambia Zimbabwe	Antigua and Barbuda Aruba Barbados Belize Canada Cape Verde Colombia Costa Rica Dominican Republic Ecuador El Salvador Guatemala Haiti Honduras Jamaica Mexico Nicaragua Panama Peru Puerto Rico Trinidad and Tobago United States Venezuela	Alabama Alaska Arizona Arkansas California Colorado Connecticut Delaware Florida Georgia US Hawaii Idaho Illinois Indiana Iowa Kansas Kentucky Louisiana Maine Maryland Massachusetts Michigan Minnesota Mississippi Missouri Montana Nebraska Nevada New Hampshire New Jersey New Mexico New York North Carolina North Dakota Ohio Oklahoma Oregon Pennsylvania Rhode Island South Carolina South Dakota Tennessee Texas Utah Vermont Virginia Washington West Virginia Wisconsin Wyoming	Argentina Bolivia Brazil Chile Paraguay Uruguay	Austria Belgium Czechia Denmark Estonia Finland France Germany Hungary Ireland Italy Latvia Liechtenstein Lithuania Luxembourg Netherlands Norway Poland Portugal Slovakia Spain Sweden Switzerland United Kingdom

**Table 6:** Covid-19 Lockdown’s Taxonomy based on three economy-related types of activities in 2020 (1. groceries & pharmacies 2. retail & recreation areas 3. workplaces)

Lockdown type	Key features	Representative case
Cyclic type lockdown	Intense few-day long lockdown reoccurring every second or third month with more than four in a year	Argentina: (Fig. 10)
(Several) Distinct type lockdown	Less than four few-day long lockdowns (randomly distributed except spring and autumn windows) with more than two in a year	Australia: (Fig. 11)
Gradual return to full activity	Single intense spring time lockdown with very slow recovery pattern throughout the entire year	Bangladesh: (Fig. 12)
Main lockdown followed by selective workplaces reduction	All types of activity lockdown followed by second selective lockdown with reduction only in the workplaces	Cameroon: (Fig. 13)
Minimal but steady reduction of activity	Except initial spring time, a more intense reduction lockdown limited mainly to workplaces with reduced activity throughout the year with mediocre intensity between 20 and 30% (compared to normal) and an additional selective autumn lockdown for retail and recreational areas	Germany: (Fig. 14) Spain: (Fig. 15)
Retail and Recreation type lockdown	Big focus on intense reduction of activity in retail and recreational areas. Workplaces lockdown limited throughout the year with significant reduction only in the main spring time lockdown. Groceries and pharmacies activity normal or even high, except spring time total lockdown	India: (Fig. 16)
Two main lockdowns with additional short drop in activity	Two main lockdowns (spring and autumn) with additional shorter randomly distributed third lockdown	Israel: (Fig. 17) Turkey: (Fig. 18)
Two main lockdowns with summer work activity decrease	Two main lockdown (spring and autumn) with additional shorter workplaces activity reduction in the summer. It is combined with extra activity in retail and recreational areas as well as groceries and pharmacies	Italy: (Fig. 19) Poland: (Fig. 20)
Single intense lockdown	Single intense spring time lockdown with almost normal activity in the rest of the year except one or two outliers which last for only few days (NZ accomplished total eradication of the virus)	Mauritius: (Fig. 21) New Zealand: (Fig. 22)
Two intense lockdowns	Two very intense lockdowns (spring and autumn) of comparable duration and intensity	Myanmar: (Fig. 23)
Summer time lockdown	Second lockdown is in the middle of the year, with overall intensity higher than in the initial spring time lockdown	Norway: (Fig. 24) Sweden: (Fig. 25)
Full year intense lockdown	Full year very intense lockdown with comparable intensity throughout the year except spring time total lockdown	Philippines: (Fig. 26)
Second half of the year lockdown	Main lockdown in the second half of the year (usually autumn) with additional minimal lockdown in the spring	Tanzania: (Fig. 27)
Full year lockdown with medium intensity	Spring time total lockdown with additional workplaces activity reduction throughout the year and medium intensity of between 35 and 40% (compared to normal) with additional full year minimal reduction of activity for retail and recreational areas	USA: (Fig. 28) Russia: (Fig. 29)
Medium intensity lockdown with unusual workplaces activity increase	Spring time total lockdown with additional selective reduction of activity for retail and recreational areas throughout the year. Workplaces activity increased in weeks prior to main lockdown and higher than normal in the following months (with normal activity in the rest of the year). This unusual activity pattern can be found only in one country or state (out of those analyzed by Google): Vietnam	Vietnam: (Fig. 30)

and Figures 10, 11, 12, 13, 14, 15, 16, 17, 18, 19, 20, 21, 22, 23, 24, 25, 26, 27, 28, 29, 30.
